# Delay or postponement of medical care among older adults in the Netherlands at earlier and later stages of the COVID-19 pandemic

**DOI:** 10.1007/s40520-022-02266-x

**Published:** 2022-10-19

**Authors:** Marlou Mizee, Laura A. Schaap, Emiel O. Hoogendijk, Natasja M. van Schoor

**Affiliations:** 1 Epidemiology and Data Science, Amsterdam UMC Location Vrije Universiteit Amsterdam, De Boelelaan 1117, Amsterdam, The Netherlands; 2 Amsterdam Public Health Institute, Aging and Later Life, Amsterdam, The Netherlands; 3 Department of Health Sciences, Faculty of Science, Vrije Universiteit Amsterdam, Amsterdam, The Netherlands

**Keywords:** COVID-19, Healthcare use, Older adults, Pandemic, Care delay

## Abstract

**Aims:**

The aim of the current study was to compare cancellations or postponement of medical care among older adults during the COVID-19 pandemic between 2021 and 2020.

**Methods:**

Data of respondents aged ≥ 62 years were used from the longitudinal aging study Amsterdam (LASA), collected in 2020 and 2021, directly after the main COVID-19 waves in the Netherlands. A questionnaire assessed cancellations of medical care and postponed help-seeking behavior. Descriptive analyses were performed.

**Results:**

Overall, cancellations declined from 35% in 2020 (sample *n* = 1128) to 17% in 2021 (sample *n* = 1020). Healthcare-initiated cancellations declined from 29 to 8%. Respondent-initiated cancellations declined from 12 to 7%. Postponed help-seeking remained around 8%.

**Conclusions:**

In 2021, less cancellations were reported compared to just after the first wave of the pandemic in 2020, while postponed help-seeking remained the same. It is important to investigate how cancellations and postponed help-seeking can be prevented in future pandemics.

## Introduction

Since the outbreak of the COVID-19 pandemic in early 2020, routine healthcare has been majorly disrupted because of governmental measures to contain the pandemic and unburden healthcare professionals [1]. Changes in healthcare utilization and accessibility of care may particularly be a concern for older adults, among whom chronic conditions and multimorbidity are highly prevalent [2, 3]. It has been suggested that the disruption of medical care, and the subsequent cancellation and postponement of care, may have severe consequences for the well-being and functioning of older adults in the short- and long-term [3].

There have been several publications on the delay or postponement of medical care during 2020 among the older population [4–7]. Overall, these studies showed a decline in healthcare utilization among older adults during the first stage of the COVID-19 pandemic compared to the period before the pandemic. In an earlier LASA publication, it was reported that during the first stage of the pandemic, 35% of the sample reported cancellation of medical care and 8% reported postponed help-seeking [8]. In an editorial, several reasons for the decrease in health care utilization were suggested, such as fear of infection, COVID-19-related reorganizations of health care leading to reduced accessibility, and shortage of healthcare professionals in non-COVID-19 care [9]. At later stages of the pandemic, several attempts were made to adapt the healthcare system to the COVID-19 situation. For example, preparations were made in setting up separate COVID-19 units, regular healthcare services were scaled up to its regular capacity and face-to-face consultations were partly converted into video and telephone consultations [1]. Due to these adaptions, it is likely that more healthcare services were able to resume their regular activities.

The aim of the current study was to compare the extent to which older adults in the Netherlands report cancellation or postponement of medical care in 2021—a later stage of the pandemic—with 2020, just after the first stage of the pandemic. It is hypothesized that due to adaptations of the Dutch healthcare system to the COVID-19 situation there were less cancellations and postponements in 2021 compared to 2020.

## Methods

### Design and participants

The study was performed as an ancillary study of LASA, an ongoing cohort study that performs measurements approximately every three years among a representative sample of older adults in the Netherlands. The current study extends a previous LASA publication from 2020 on the same topic [8]. A first COVID-19 questionnaire was sent to 1485 eligible respondents in June 2020, after the first COVID-19 wave, of which 1128 participated (response: 76%). A second questionnaire was sent to 1325 eligible respondents in March 2021, after the second COVID-19 wave, of which 1020 participated (response: 77%). The overlapping sample consisted of 993 respondents. Details on the sampling, measurements and data collection of the LASA COVID-19 study have been published previously [10]. All respondents signed a written informed consent and the study was approved by the medical ethics committee of the VU University Medical Center.

### Cancellations and postponed help-seeking

Cancellation of medical care was measured by asking the respondents whether they had an appointment cancelled (i.e., primary care, outpatient specialist) either initiated by the healthcare professional or initiated by themselves since the beginning of the COVID-19 pandemic (first questionnaire, 2020) or since October 2020 (second questionnaire, 2021). In addition, we asked how many planned visits were replaced by a telephone consult in 2020 and by a telephone or video consult in 2021. Physical therapist visits were only included in the 2021 questionnaire and, therefore, presented separately. Postponed help-seeking was measured by asking the respondents if, due to the COVID-19 situation, they did not seek the necessary help in case of physical or mental health problems. In 2021, we asked about the reasons for the postponement. Compared to our first publication on care delay during the COVID-19 pandemic [8], the numbers in the current paper vary slightly. In the previous publication, only complete cases with data on various health characteristics were included, while for the current paper we included all participants [8].

### Characteristics of respondents

Data on age and sex were derived from population registries. Educational level was assessed by asking the respondent for the highest education level completed. This was converted into elementary school or less (low), secondary school (medium) or higher education (high). Number of chronic diseases was measured through self-reported presence of the following chronic diseases: chronic obstructive pulmonary disease, cardiac disease, peripheral arterial disease, diabetes mellitus, stroke, cancer, and joint disorders (osteoarthritis/rheumatoid arthritis). Functional limitations were assessed using self-reported questions on the following activities: walking up and down a staircase of 15 steps without resting, dressing and undressing oneself, sitting down and standing up from a chair, cutting one’s own toenails, walking outside for five minutes without resting, taking a bath or shower, and using own or public transportation. We distinguished the following functional limitations categories: no limitations, mild limitations (having difficulty with at least one activity) and severe functional limitations (respondent could not perform at least one activity or only with help). Depressive symptoms were assessed using the 10-item version of the center for epidemiologic studies depression scale (CES-D-10) and were summed to a total score ranging from 0 to 30. A cut-off score of ≥ 10 indicated the presence of clinically relevant depressive symptoms [11]. Anxiety symptoms were measured using the anxiety subscale of the Hospital Anxiety and depression scale (HADS-A) and summed to a total score ranging from 0 to 21. A cut-off score of ≥ 8 was applied to indicate the presence of clinically significant anxiety symptoms [12].

### Statistical analysis

Descriptive analyses were performed to report the characteristics of respondents participating in the 2020 and 2021 questionnaires, respectively. In addition, the percentage of cancellations, either healthcare- or respondent-initiated cancellations, or postponement of medical care, both in 2020 and 2021, were described. Analyses were done in SPSS 26 (IBM Corp, Armonk, NY, USA).

## Results

**Fig. 1:**
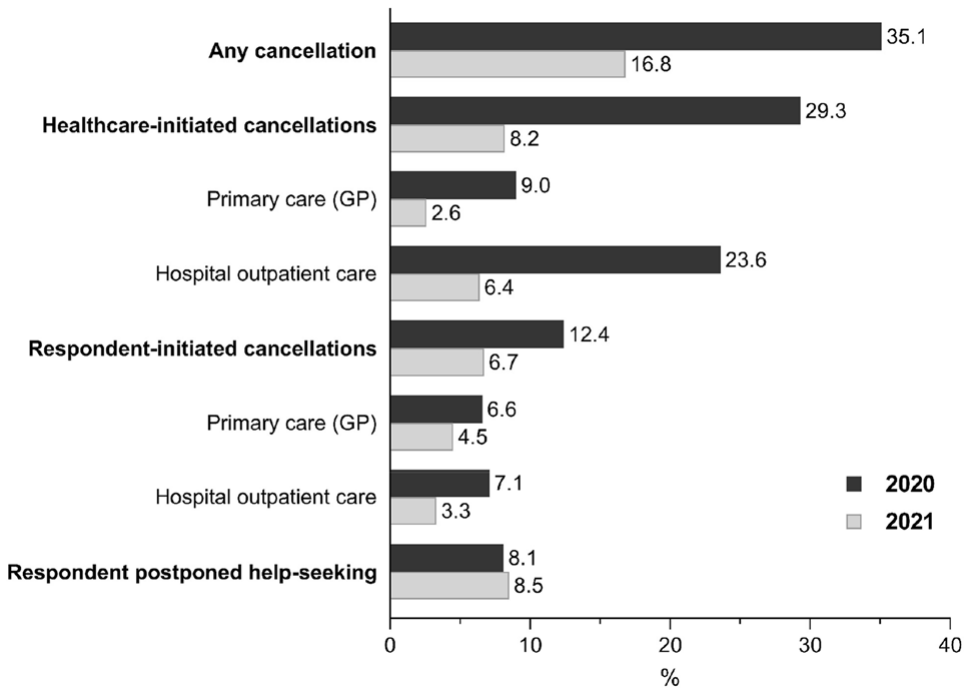
Cancellation and postponement of medical care among older adults in the Netherlands during the COVID-19 pandemic: comparison between 2020 and 2021

Characteristics of the samples from 2020 to 2021 are provided in Table 1. Mean age was 74 years and about 52% was female both in 2020 and in 2021. In Fig. 1, the percentages of cancellations and postponements stratified by healthcare setting are shown for 2020 and 2021. The total percentage of cancellations was lower in 2021 (16.8%) as compared to 2020 (35.1%). Healthcare-initiated cancellations were reported by 29.3% of respondents in 2020 and by 8.2% in 2021. In 2020, 15.4% of participants indicated that their planned visit to the general practitioner was replaced by a telephone consult, while in 2021 8.7% of respondents reported that their visit was replaced by a telephone or video consult. For the medical specialist, this percentage decreased from 21.3% in 2020 to 16.5% in 2021. Respondent-initiated cancellations were reported by 12.4% of respondents in 2020 and by 6.7% in 2021. Postponed help-seeking changed from 8.1% in 2020 to 8.5% in 2021. In 2021, we asked about the reasons for the postponement. Out of 87 respondents who postponed help-seeking, 28 persons did not want to burden the care system, 25 were afraid to get infected with COVID-19, 7 were afraid to infect others and 23 reported other reasons, such as having COVID-19, having a cold or having another health problem, being in quarantine or waiting for the test result, having a partner at risk for COVID-19, and transportation problems. Furthermore, in 2021, 4.2% reported a healthcare-initiated physiotherapy cancellation and 2.5% reported a respondent-initiated physiotherapy cancellation.

**Table 1:** Characteristics of the study samples in 2020 and 2021

Characteristics	2020 sample *n* = 1128	2021 sample *n* = 1020
	Mean (SD) or *n* (%)	Mean (SD) or *n* (%)
Age, 62–103 years, mean (SD)	73.9 (7.5)	74.2 (7.1)
Sex, female, *n* (%)	596 (52.8)	534 (52.4)
Educational level		
Low, *n* (%)	132 (11.7)	104 (10.2)
Medium, *n* (%)	380 (33.7)	340 (33.3)
High, *n* (%)	616 (54.6)	576 (56.5)
Chronic diseases, 0–7, mean (SD)	1.3 (1.1)	1.3 (1.1)
Functional limitations		
No limitations, *n* (%)	602 (54.3)	533 (52.7)
Mild, *n* (%)	327 (29.5)	304 (30.0)
Severe, *n* (%)	180 (16.2)	175 (17.3)
Depressive symptoms, CES-D-10 ≥ 10, *n* (%)	199 (18.0)	165 (16.3)
Anxiety symptoms, HADS-A ≥ 8, *n* (%)	115 (10.5)	79 (7.8)

*n* may vary across covariates due to missing data

## Discussion

The results of this study show to what extent older adults in the Netherlands reported cancellations or postponement of medical care during earlier and later stages of the COVID-19 pandemic. The results indicate that in 2021, 16.8% of the sample reported any cancellation. This is half of what was reported in 2020 (35.3%). The percentage reported postponed help-seeking did not markedly change between 2020 and 2021.

When comparing the results with previous studies among older populations, it should be noted that, to our knowledge, no previous publications have reported on a comparison of healthcare use in different periods during the pandemic. Several publications showed a decrease in the use of healthcare services in the first stages of the COVID-19 pandemic compared to pre-pandemic healthcare use, in the general population and in older populations [4–8]. A study from the UK showed a decline of 70% in the utilization of healthcare services during the first months of the pandemic compared to before the pandemic. This study also showed that in August 2020 healthcare utilization increased steadily and approached pre-pandemic rates [7]. A study from Singapore showed the same pattern, with a decline in the number of medical visits by older adults during the first stage of the pandemic, followed by an increase that reached the regular pre-COVID-19 medical visit numbers at the end of 2020 [13]. In the Netherlands, the Dutch Healthcare Authority revealed that the number of healthcare referrals were since mid-April 2021—1 month after sending out our second questionnaire—around 96% of the expected number of referrals if the COVID-19 pandemic had not taken place [14].

Despite the fewer reported cancellations in later stages of the pandemic, cancellations are still reported. It was suggested that due to the adaption of the healthcare system to the COVID-19 situation, regular healthcare services were more able to continue healthcare services. This suggestion is supported by data from our study, as healthcare-initiated cancellations showed a larger decrease (from 29.3% in 2020 to 8.2% in 2021) compared to respondent-initiated cancellations (from 12.4% in 2020 to 6.7% in 2021). In our first COVID-19 questionnaire we asked about cancellations that occurred between the start of the pandemic until June/July 2020. During this first stage of the pandemic, a lockdown was in effect during the first few months, but many COVID-19 measures were lifted in June. In our second COVID-19 questionnaire we asked about cancellations that occurred between October 2020 and March 2021. During that time period, the number of COVID-19 infections was rising again, leading to a second COVID-19 wave, which was followed by a lockdown of the country in November 2020 that lasted until March 2021. By that time, a few older adults had been vaccinated. Therefore, although we are comparing different stages of the pandemic, some similarities exist, including the high number of infections and the lockdown of the country during both stages of the pandemic. Part of the cancellations that are still present in 2021 could therefore be due to a COVID-19 infection (of either the respondent or the health care professional) or due to consequences of the lockdown, e.g. public transportation limitations. Interestingly, the percentage of planned visits that was replaced by a telephone or video consult decreased between 2020 and 2021. As there were less cancellations in 2021 as compared with 2020, telephone or video consults may not have been necessary anymore in 2021 for part of the consults. It is important to investigate how cancellations and postponed help-seeking can be prevented or minimized in future pandemics to continue health services and prevent potential negative health outcomes. Detailed monitoring of cancellations and postponed help-seeking behavior may help policymakers to make the healthcare systems more resilient in current and future pandemics. Additionally, a longitudinal follow-up of health outcomes is needed to better understand the possible consequences of cancellations and postponed help-seeking behaviour.

A strength of this study is the use of data from a large, representative sample of community-dwelling older adults. To our knowledge, this is the first study which compared the reported cancellation and postponed help-seeking between different stages of the COVID-19 pandemic. However, there are some limitations to this study. At both time moments, cancellation and postponement of healthcare were based on self-report, which might have led to under- or over-reporting due to recall bias. Additionally, the data does not include details on the medical appointments, and the severity of the health issue for which an appointment was made. Furthermore, our results concern the older population in the Netherlands and it remains unknown to what extent results can be generalized to other countries with a different healthcare system and other policies concerning COVID-19.

To conclude, fewer cancellations were reported in 2021 when compared to 2020. The highest decrease was observed in healthcare-initiated cancelations, which suggests adaptation of the healthcare system to the COVID-19 situation. Nonetheless, cancellations and postponed help-seeking are still reported. It is important to investigate how these cancellations and postponed help-seeking can be prevented in future pandemics.

## Data Availability

The dataset generated during the current study are not publicly available due to confidentiality, but the data underlying the results presented in this study are available from the longitudinal aging study Amsterdam (LASA). Data from LASA, including data from the LASA COVID-19 questionnaire, may be requested for research purposes. More information on data requests can be found on the LASA website: http://www.lasa-vu.nl.
